# Acute Submaximal Running Potentiates the Straub Tail Response to Meperidine in a Dose- and Sex-Dependent Manner in Mice

**DOI:** 10.1021/acsptsci.6c00328

**Published:** 2026-08-31

**Authors:** Tatyana Nery, Naiara de Souza Santos, Ana Cristina de Bem Alves, Amanda Leite Bastos-Pereira, Aderbal S. Aguiar

**Affiliations:** Biology of Exercise Lab (Labioex), Department of Physical Therapy, UFSC-Federal University of Santa Catarina, Araranguá, SC 88905-120, Brazil

**Keywords:** behavioral pharmacology, exercise-induced hypoalgesia, nociception, opioid potentiation, pethidine

## Abstract

Physical exercise recruits endogenous opioid mechanisms in the central nervous system, but whether this physiological opioid tone potentiates the effects of exogenous μ-agonists, and whether such interactions differ between sexes, remains unknown. Here, we used the Straub tail response as a behavioral index of central μ_2_-opioid receptor activity to investigate whether acute submaximal running modulates the pharmacodynamic effects of meperidine in male and female Swiss mice. In resting animals, meperidine produced a dose- and time-dependent Straub tail response across a 12-fold dose range, and the submaximal doses of 8 and 12 mg/kg were selected for treadmill experiments. Running at approximately 70% of V̇O_2_ max potentiated the Straub tail response at 12 mg/kg in both sexes, with the effect substantially larger in females, as indicated by a significant Sex × Exercise interaction. Thermal antinociception was equivalent between sexes at 12 mg/kg, indicating that the dimorphism was specific to the exercise-opioid interaction rather than to baseline drug sensitivity. A separate experiment testing whether running alone elicits a naloxone-sensitive response was inconclusive in both sexes; under opioid receptor blockade, however, females maintained higher running-induced scores than males, indicating that the sex difference persists during naloxone treatment. These findings indicate that acute submaximal running potentiates the Straub tail response to meperidine in a dose- and sex-dependent manner. The sexually dimorphic nature of this interaction suggests that biological sex and exercise intensity may jointly modulate opioid pharmacodynamics during physical activity.

## Graphical abstract

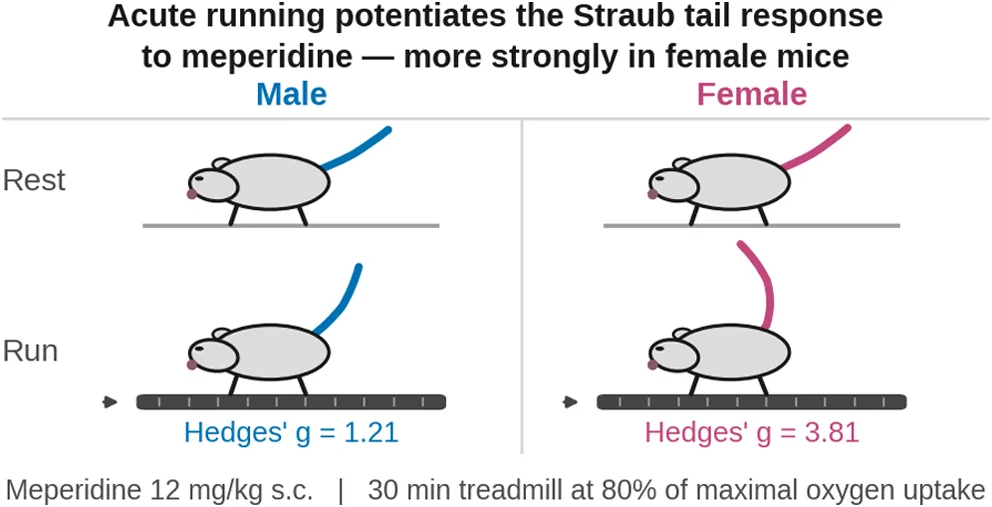

Physical exercise produces a broad spectrum of physiological and psychological effects, many of which are mediated by the endogenous opioid system. The euphoric states experienced during and after prolonged running, commonly described as the runner’s high, are characterized by anxiolysis, analgesia, sedation, and euphoria, and have long been attributed to exercise-induced endorphin release.^1^ This hypothesis originates in classical human studies showing that exercise increases circulating β-endorphin and β-lipotropin, an effect facilitated by physical conditioning,^2,3^ and that naloxone reverses the postrun elevation of ischemic, but not thermal, pain thresholds after long-distance running,^4^ a literature critically reviewed by Sforzo.^5^ These early studies, however, relied on peripheral hormone measurements and psychophysical end points and could not establish a central site of action. The first neurobiological evidence in humans came from Boecker et al.,^6^ who used positron emission tomography to measure region-specific reductions in opioid receptor availability in prefrontal and limbic brain structures of endurance runners after 2 h of running, with radioligand binding correlating inversely with the level of perceived euphoria. Because PET quantifies the availability of opioid receptors to a radioligand rather than the release of endogenous peptides, this finding provides indirect, though regionally specific, evidence of central opioid neurotransmission during running in humans.

The endogenous opioid system, defined pharmacologically by the identification of the opiate receptor^7^ and of its endogenous peptide ligands,^8^ provides the substrate for these effects. In rodents, the central opioidergic response to exercise has been characterized at the neurochemical and receptor levels. Regular treadmill training increases β-endorphin expression in multiple central nuclei in an intensity-dependent manner;^9^ acute forced or voluntary exercise upregulates μ-opioid receptor expression in the hippocampal formation;^10^ and regular training elevates endogenous opioid content in the periaqueductal gray and rostral ventromedial medulla, key nodes of the descending pain modulatory system, with antinociceptive effects reversed by systemic naloxone.^11^ Collectively, these findings support the view that submaximal aerobic exercise recruits endogenous μ-opioid mechanisms in the central nervous system sufficient to produce measurable behavioral and pharmacological effects.

The pharmacological consequences of this exercise-induced opioid tone remain incompletely understood, particularly regarding interactions with exogenous opioid drugs. If running elevates central μ-opioid tone, it should, in principle, potentiate the effects of exogenously administered μ-agonists, an interaction of potential clinical interest, since current guidance for patients already receiving opioid therapy recommends optimizing nonopioid approaches, with exercise and exercise therapy prominent among them, while opioid treatment continues.^12^ The Straub tail response in mice offers a sensitive and pharmacologically specific behavioral index to test this hypothesis. First described by Straub^13^ as a highly sensitive biological reaction to morphine in mice, and anatomically attributed to spastic contraction of the sacrococcygeus dorsalis muscle,^14^ the response was subsequently characterized as opioid-specific and antagonist-reversible.^15,16^ The response is mediated by central μ_2_-opioid receptors, dissociable from μ_1_-mediated analgesia,^17^ and can be induced not only by exogenous opioids but also by endogenous β-endorphin when this peptide is sufficiently elevated by a nonpharmacological stimulus: in mice, skin-derived β-endorphin induced by localized ionizing radiation produced both Straub tail and naloxone-reversible thermal antinociception, effects that were absent in β-endorphin-null animals.^18^ This makes the response an ideal readout of additive μ-opioid engagement.

Sex differences in opioid pharmacology add a further dimension of complexity. Clinical and preclinical evidence indicates that males and females differ in their sensitivity to opioid analgesics, in the circuits engaged during exercise-induced pain modulation, and in the degree to which endogenous opioids mediate exercise-induced hypoalgesia.^19–22^ Whether these differences extend to the interaction between exercise and exogenous opioids, and whether they reflect receptor-level or circuit-level dimorphism, remains unknown.

The present study was designed to address these gaps. Our objective was to determine whether acute submaximal running modulates the pharmacodynamic response to a systemically administered μ-opioid agonist in mice, and whether this modulation is sex-dependent. Meperidine was selected as the μ-agonist on pharmacodynamic grounds: it is a phenylpiperidine, structurally distinct from the morphinans, whose comparatively low potency affords a broad submaximal dose range, and whose high lipid solubility confers a rapid onset and a duration of action compatible with a 30 min running session; it is also one of the opioids for which the Straub tail response was originally characterized, by subcutaneous administration.^15^ Using the Straub tail response as a behavioral readout of central μ-opioid activity, we first characterized the dose–response and time course of the drug at rest in order to identify submaximal doses, and then addressed three questions: (i) whether acute submaximal running potentiates the Straub tail response induced by those doses; (ii) whether running alone elicits a naloxone-sensitive Straub-like response, which would indicate an endogenous opioid contribution; and (iii) whether the magnitude of the exercise-opioid interaction differs between male and female mice. The results indicate a dose- and sex-dependent interaction between acute running and the behavioral effects of meperidine, the mechanism of which is not resolved by the present design.

## Results and Discussion

### Meperidine Produces a Dose-Dependent Straub Tail Response in Resting Mice

Meperidine produced a dose- and time-dependent Straub tail response in resting mice of both sexes, consistent with central μ-opioid activity at the doses tested. As noted above, the response indexes central μ_2_-opioid receptor activity,^17^ and provides a sensitive behavioral readout of supraspinal μ-opioid receptor engagement.^23^ In resting male mice, a significant dose × time effect was observed across the 60 min observation period (*F*(76, 665) = 6.58, η^2^ *p* = 0.429, 1 – β = 1.00, *P* < 0.0001; Figure 1A), with Bonferroni post hoc analysis confirming that all meperidine-treated groups differed from vehicle. An analogous dose- and time-dependent pattern was observed in female mice (*F*(76, 665) = 4.84, η^2^ *p* = 0.356, 1 – β = 1.00, *P* < 0.0001; Figure 2A), with all doses differing from vehicle after correction. Based on these profiles, 8 and 12 mg/kg were selected as submaximal doses, producing measurable but nonmaximal responses, to create a pharmacodynamic window within which an additional effect of exercise could be detected. That such a window could be defined at all reflects the choice of agonist: meperidine produced graded, nonsaturating responses across a 12-fold dose range (8–96 mg/kg; Figures 1A and 2A). This reproduces the original characterization of the drug in this assay, in which meperidine ranked below morphine in potency across rodent and human analgesic measures and failed to reach a 100% response rate even at 64 mg/kg.^15^ A higher-potency μ-agonist would compress this range and leave little room for an additive endogenous contribution to be resolved.

**Figure 1:**
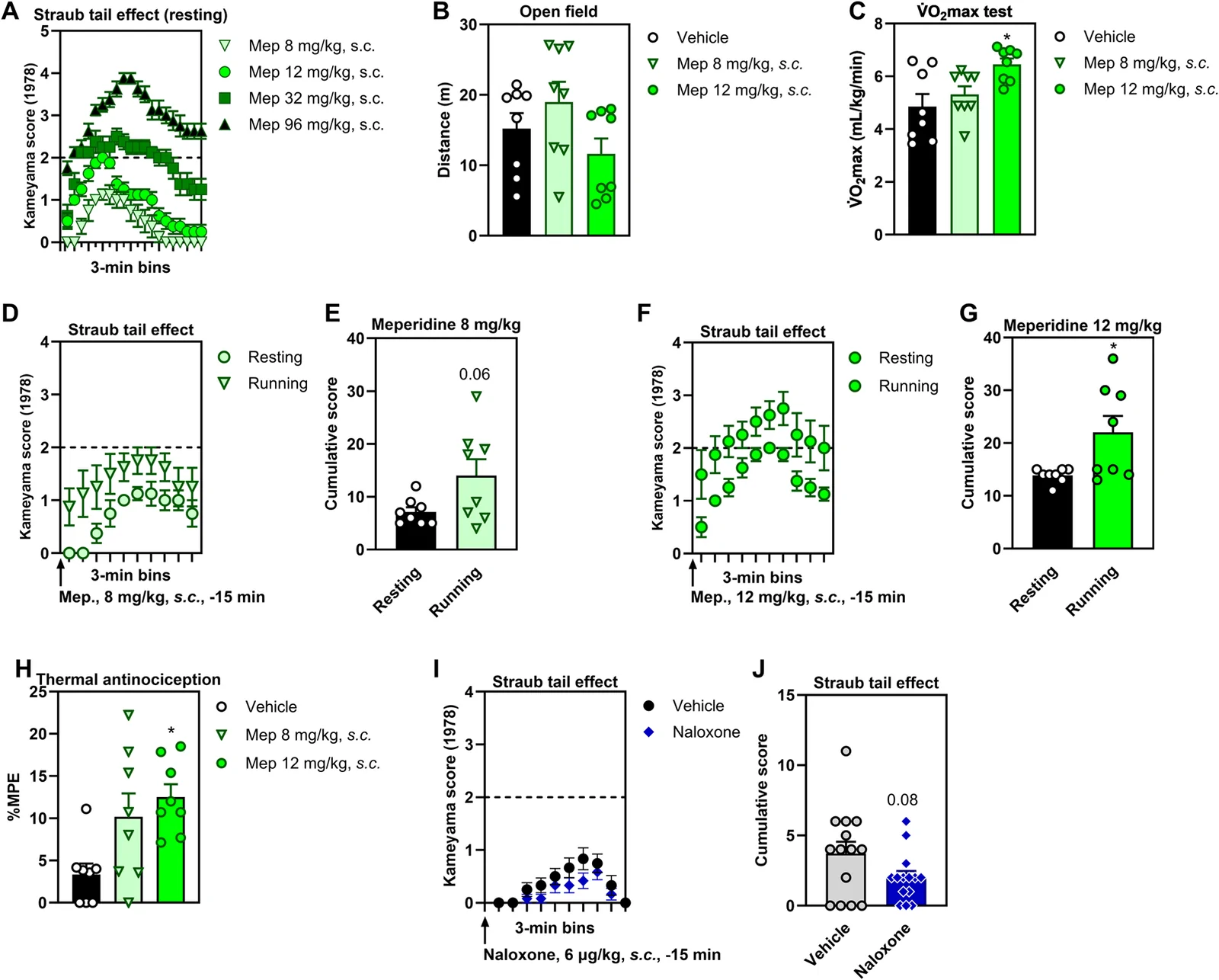
Meperidine induces a dose- and time-dependent Straub tail response that is potentiated by running in male mice. Panels A–C characterize the pharmacological and physiological model. Panel A shows that meperidine dose-dependently increased Straub tail scores in resting mice, with the highest and most persistent response at 96 mg/kg and a mild, transient response at 8 mg/kg. Panel B shows that meperidine did not alter spontaneous locomotor activity at 8 or 12 mg/kg. Panel C shows that 12 mg/kg meperidine increased V̇O_2_max compared with vehicle, whereas 8 mg/kg did not. Panels D–H show the effects of running on meperidine-induced Straub tail behavior and thermal antinociception. Panels D and F show the time course of the Straub tail response during a 30 min running session in resting and running mice at 8 and 12 mg/kg; the group × time interaction was not significant at either dose, and the main effect of group was significant at 12 mg/kg. Panels E and G show that cumulative scores were higher in running mice, reaching significance at 12 mg/kg. Panel H shows that thermal antinociception increased only at 12 mg/kg meperidine. Panels I and J show the effect of naloxone on the running-induced Straub-like response. Panel I shows the time course of the Straub tail response during running after vehicle or naloxone (6 μg/kg, s.c.); neither the main effect of treatment nor the group × time interaction was significant. Panel J shows the corresponding cumulative scores, which did not differ significantly between groups (*P* = 0.079, one-tailed). Data are presented as mean ± SEM; *n* = 8 animals per group for panels A–H and *n* = 12 per group for panels I and J. Time-course data (A, D, F, I) were analyzed by mixed ANOVA or two-way repeated-measures ANOVA. In panel A, the vehicle group is not visible because every vehicle-treated animal scored zero at every observation; all four meperidine doses differed from it after Bonferroni post hoc correction. Cumulative scores (E, G, J) and group comparisons (B, C, H) were analyzed by Welch’s unpaired *t* test or one-way ANOVA followed by Tukey’s post hoc test, as appropriate. *P < 0.05 vs vehicle or resting condition. The dashed line in panels A, D, F, and I indicates a Kameyama score of 2.

**Figure 2:**
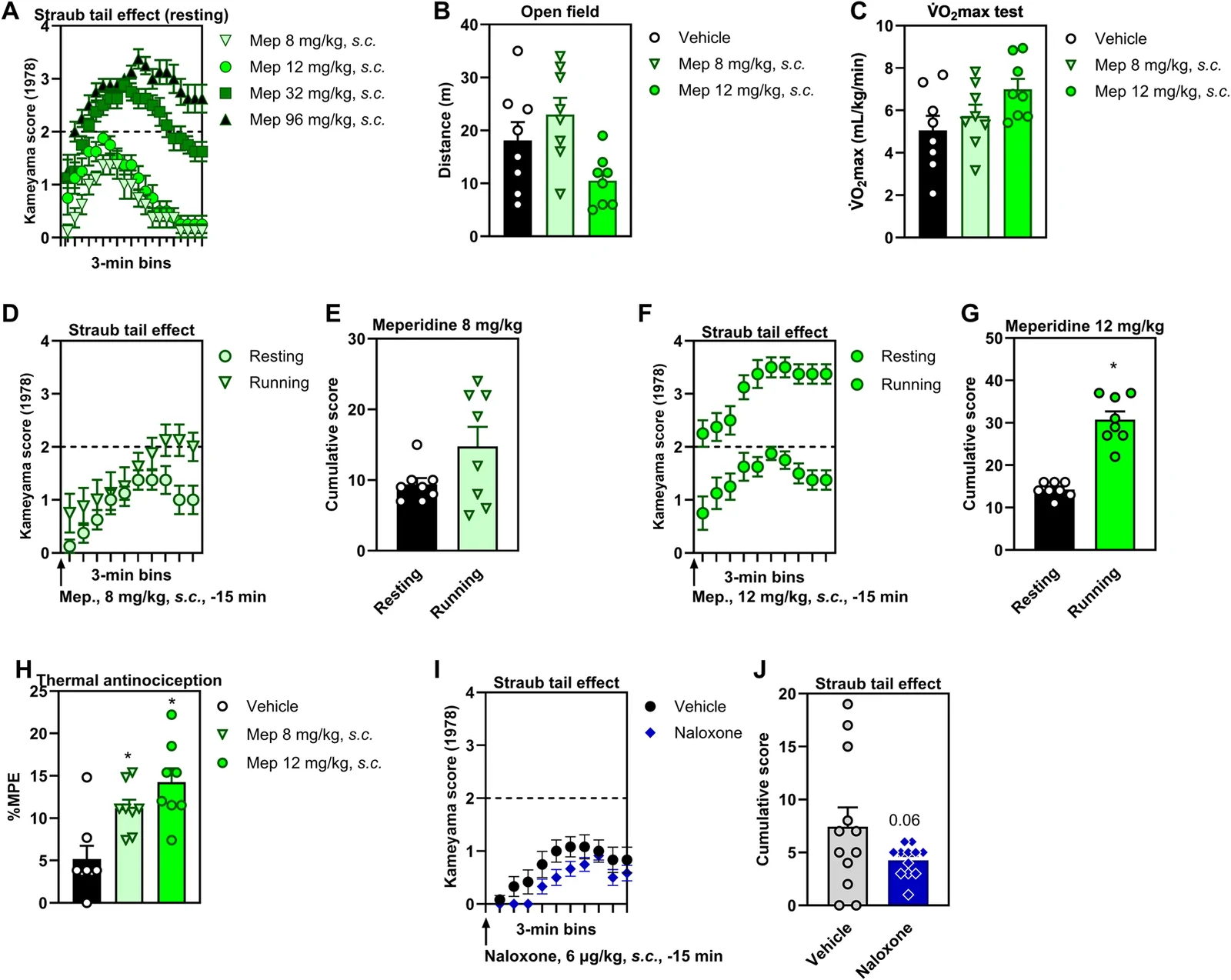
Meperidine induces a dose- and time-dependent Straub tail response in female mice that is potentiated by running at 12 mg/kg. Panel A shows that meperidine dose-dependently increased Straub tail scores in resting female mice, with the strongest and most sustained response at 96 mg/kg and a mild, transient response at 8 mg/kg. Panel B shows that meperidine did not alter spontaneous locomotor activity compared with vehicle at either dose. Panel C shows that neither dose significantly altered V̇O_2_max. Panels D–H show the effects of running on meperidine-induced Straub tail behavior and thermal antinociception. Panels D and F show the time course of the Straub tail response during a 30 min running session in resting and running mice after meperidine at 8 and 12 mg/kg, respectively. Panels E and G show the corresponding cumulative Straub tail scores; running significantly increased cumulative scores at 12 mg/kg. Panel H shows that thermal antinociception increased at both 8 and 12 mg/kg relative to vehicle. Panels I and J show the effect of naloxone on the running-induced Straub-like response. Panel I shows the time course of the Straub tail response during running after vehicle or naloxone (6 μg/kg, s.c.). Panel J shows the corresponding cumulative Straub tail scores; naloxone did not significantly reduce scores compared with vehicle (*P* = 0.060, one-tailed). Data are presented as mean ± SEM; *n* = 8 animals per group for panels A–H and *n* = 12 per group for panels I and J. Time-course data (A, D, F, I) were analyzed by mixed ANOVA or two-way repeated-measures ANOVA. In panel A, the vehicle group is not visible because every vehicle-treated animal scored zero at every observation; all four meperidine doses differed from it after Bonferroni post hoc correction. Cumulative scores (E, G, J) and group comparisons (B, C, H) were analyzed by Welch’s unpaired *t* test or one-way ANOVA followed by Tukey’s post hoc test, as appropriate. *P < 0.05 vs vehicle or resting condition. The dashed line in panels A, D, F, and I indicates a Kameyama score of 2.

Meperidine did not alter spontaneous locomotor activity at either dose in males (*F*(2, 21) = 2.27, η^2^ *p* = 0.178, 1 – β = 0.41, *P* = 0.128; Figure 1B) or females (*F*(2, 21) = 4.98, η^2^ *p* = 0.322, 1 – β = 0.75, *P* = 0.017; Figure 2B); the omnibus effect in females arose from a difference between the two meperidine doses (Tukey post hoc: 8 vs 12 mg/kg, *P* = 0.013), neither of which differed from vehicle (*P* = 0.443 and *P* = 0.166). Neither dose therefore altered locomotion relative to vehicle in either sex, indicating that the Straub tail response was not accompanied by generalized hyperlocomotion and that subsequent potentiation during running is unlikely to reflect nonspecific motor activation. Cardiopulmonary exercise testing confirmed that the running speed of 20 m/min corresponded to 72 ± 15% of V̇O_2_ max in males and 67 ± 15% in females, an intensity within the range over which central opioid changes have been reported in rodents.^9,11^ At this intensity, 12 mg/kg meperidine increased V̇O_2_ max in males (*F*(2, 21) = 5.53, η^2^ *p* = 0.345, 1 – β = 0.80, *P* = 0.012; Figure 1C) but not in females (*F*(2, 21) = 2.91, η^2^ *p* = 0.217, 1 – β = 0.51, *P* = 0.077; Figure 2C), suggesting sex-dependent cardiovascular interactions at this dose, whose bearing on the behavioral end points is considered below.

### Running Potentiates the Meperidine-Induced Straub Tail Response

Running at a submaximal intensity potentiated the Straub tail response induced by submaximal doses of meperidine in both sexes, an effect that would be consistent with exercise-induced recruitment of central endogenous opioid tone summating with the pharmacological effects of the exogenous μ-agonist, although the present experiments do not isolate that mechanism. Such an interpretation would be compatible with evidence that acute exercise engages central opioid mechanisms: indirectly in humans, through reduced opioid receptor availability on PET,^6^ and directly in rodents, through increased central β-endorphin expression,^9^ and with the observation that endogenous β-endorphin, elevated in mice by a nonpharmacological stimulus, produced both Straub tail behavior and naloxone-reversible thermal antinociception.^18^

In male mice, running increased Straub tail scores relative to resting throughout the 30 min session at 12 mg/kg. Mixed ANOVA on the time course revealed a significant main effect of group at 12 mg/kg (*F*(1, 14) = 6.50, η^2^ *p* = 0.317, 1 – β = 0.66, *P* = 0.023; Figure 1F) and a main effect of time at both doses (8 mg/kg: *F*(9, 126) = 10.76, η^2^ *p* = 0.434, *P* < 0.0001; 12 mg/kg: *F*(9, 126) = 14.76, η^2^ *p* = 0.513, *P* < 0.0001). The group × time interaction was not significant at either dose (8 mg/kg: *F*(9, 126) = 1.22, η^2^ *p* = 0.080, 1 – β = 0.58, *P* = 0.289; 12 mg/kg: *F*(9, 126) = 0.39, η^2^ *p* = 0.027, 1 – β = 0.19, *P* = 0.937), indicating that the difference between running and resting animals did not vary systematically across the session; we therefore make no claim about the timing of the effect within the session. At 8 mg/kg, the main effect of group did not reach significance (*F*(1, 14) = 4.54, η^2^ *p* = 0.245, 1 – β = 0.51, *P* = 0.051). Cumulative scores were significantly increased by running only at 12 mg/kg (*t* = 2.55, df = 7.32, Hedges’ *g* = 1.21, 1 – β = 0.60, *P* = 0.037; Figure 1G), while the effect at 8 mg/kg, though large, did not reach significance (*t* = 2.13, df = 8.10, Hedges’ *g* = 1.01, 1 – β = 0.47, *P* = 0.065; Figure 1E). Operationally, this indicates that the effect of running became detectable only above a threshold dose of the exogenous agonist; at 8 mg/kg the added component was not large enough to shift cumulative scores. We did not measure receptor occupancy, and the present data do not speak to the receptor-level basis of this threshold. Consistent with a dose threshold, thermal antinociception on the hot plate was observed only at 12 mg/kg in males (*F*(2, 21) = 5.85, η^2^ *p* = 0.358, 1 – β = 0.82, *P* = 0.010; Tukey post hoc: 12 mg/kg vs vehicle, *P* = 0.009; Figure 1H), identifying this dose as the one at which μ_1_-mediated analgesia and μ_2_-mediated Straub potentiation were both detectable.

In female mice, running also potentiated the Straub tail response at 12 mg/kg, and did so with a substantially larger effect than in males. The main effect of group was large (*F*(1, 14) = 64.88, η^2^ *p* = 0.823, 1 – β = 1.00, *P* < 0.0001; Figure 2F), with running females showing higher scores than resting females across the session; as in males, the group × time interaction was not significant (*F*(9, 126) = 1.19, η^2^ *p* = 0.078, 1 – β = 0.57, *P* = 0.306). Cumulative potentiation was correspondingly larger than in males (*t* = 8.05, df = 8.40, Hedges’ *g* = 3.81, 1 – β = 1.00, *P* = 3.2 × 10^–5^; Figure 2G). At 8 mg/kg in females the effect was large but statistically inconclusive (main effect of group *F*(1, 14) = 3.37, η^2^ *p* = 0.194, 1 – β = 0.40, *P* = 0.088; group × time interaction *F*(9, 126) = 1.64, η^2^ *p* = 0.105, 1 – β = 0.74, *P* = 0.112; cumulative *t* = 1.84, df = 8.47, Hedges’ *g* = 0.87, *P* = 0.101; Figure 2E). In contrast to males, both 8 and 12 mg/kg meperidine produced significant thermal antinociception in females (*F*(2, 21) = 10.38, η^2^ *p* = 0.497, 1 – β = 0.97, *P* = 0.0007; Tukey post hoc: 8 mg/kg vs vehicle, *P* = 0.020; 12 mg/kg vs vehicle, *P* = 0.0006; Figure 2H), indicating a lower antinociceptive threshold in females for this μ-agonist; sex differences in opioid antinociception are strain-dependent in outbred rodents, with all directions reported.^21^

### Running and Endogenous Opioids: The Naloxone Experiment

To determine whether running itself recruits an endogenous opioid component independently of exogenous drugs, male and female mice received naloxone or vehicle before running without meperidine. This experiment did not yield a statistically significant effect of naloxone in either sex, and we present it as inconclusive rather than as evidence either for or against an endogenous opioid contribution to the running-induced behavior.

In male mice, naloxone-treated animals showed numerically lower Straub-like scores than vehicle-treated animals across the running session, but neither the main effect of treatment (*F*(1, 22) = 2.17, η^2^ *p* = 0.090, 1 – β = 0.29, *P* = 0.155) nor the group × time interaction (*F*(9, 198) = 0.95, η^2^ *p* = 0.042, 1 – β = 0.47, *P* = 0.479; Figure 1I) reached significance. The cumulative score was likewise not significantly reduced (*t* = 1.47, df = 17.24, Hedges’ *g* = 0.58, 1 – β = 0.29, *P* = 0.079 one-tailed, *P* = 0.158 two-tailed; Figure 1J).

In female mice the pattern was the same: no significant main effect of treatment (*F*(1, 22) = 2.81, η^2^ *p* = 0.113, 1 – β = 0.36, *P* = 0.108) and no group × time interaction (*F*(9, 198) = 0.78, η^2^ *p* = 0.034, 1 – β = 0.38, *P* = 0.638; Figure 2I), with the cumulative score not reaching significance (*t* = 1.68, df = 12.19, Hedges’ *g* = 0.66, 1 – β = 0.34, *P* = 0.060 one-tailed, *P* = 0.119 two-tailed; Figure 2J). The effect sizes in the two sexes were comparable and of moderate magnitude (Hedges’ *g* = 0.58 in males and 0.66 in females), and the achieved power was low in both (1 – β = 0.29 and 0.34). This experiment is therefore underpowered to detect an effect of the size observed within either sex. Considered across both sexes, however, the two-way analysis reported below does yield a main effect of treatment (*F*(1, 44) = 4.82, η^2^ *p* = 0.099, 1 – β = 0.57, *P* = 0.033), consistent with a small reduction of the running-induced response by naloxone. We report this rather than omit it, but we do not build on it: an effect that emerges only when the sexes are pooled, in an experiment whose within-sex comparisons are underpowered and whose two one-tailed cumulative comparisons both fall short of significance, is a weak basis for a mechanistic claim. Adequately powered replication within each sex would be required before the running-induced response could be described as opioid-dependent.

Two considerations follow for the interpretation of the potentiation reported above. First, the naloxone experiment tested whether running alone elicits a Straub-like response; it did not test whether the potentiation of meperidine by running is opioid-dependent. The latter would require naloxone to be administered together with meperidine before running, a condition not included in the present design. Even a clear outcome in the experiment as performed would therefore have provided only indirect evidence about the mechanism of potentiation. Second, a nonopioid explanation must be considered. Meperidine was administered subcutaneously, and running increases cutaneous and skeletal muscle blood flow; accelerated absorption from the injection site during exercise could raise plasma meperidine concentrations and produce a larger Straub tail response without any additional endogenous opioid contribution. The observation that 12 mg/kg meperidine increased V̇O_2_max in males (Figure 1C) indicates that drug and exercise do interact systemically in this preparation. Distinguishing these possibilities requires two experiments that we did not perform: opioid antagonism applied against the potentiation itself, and measurement of plasma meperidine concentrations in resting and running animals. Until these are available, the present data indicate that acute running modifies the behavioral response to a μ-agonist in a dose- and sex-dependent manner, but do not identify the mechanism of that modification.

### Sex Differences in Exercise-Opioid Interactions

The formal comparison between sexes revealed a significant Sex × Exercise interaction on cumulative Straub tail scores at 12 mg/kg meperidine (*F*(1, 28) = 4.89, η^2^ *p* = 0.149, 1 – β = 0.57, *P* = 0.035; Figure 3A), indicating that running potentiates the Straub tail response to meperidine more robustly in females than in males. This dimorphism was specific to the exercise-opioid interaction: a two-way ANOVA on hot plate %MPE revealed no Sex × Dose interaction (*F*(1, 28) = 0.00, η^2^ *p* < 0.001, 1 – β = 0.05, *P* = 0.974; Figure 3B), with equivalent antinociceptive responses to 12 mg/kg in both sexes (males: Hedges’ *g* = 2.15, *P* = 0.0014; females: Hedges’ *g* = 1.89, *P* = 0.0039; Bonferroni-corrected). The dissociation between a significant Sex × Exercise interaction in the Straub tail and the absence of a Sex × Dose interaction in thermal antinociception indicates that the dimorphism does not reflect a difference in baseline μ-opioid sensitivity to meperidine, but specifically in the capacity of exercise to potentiate the behavioral response to it.

**Figure 3:**
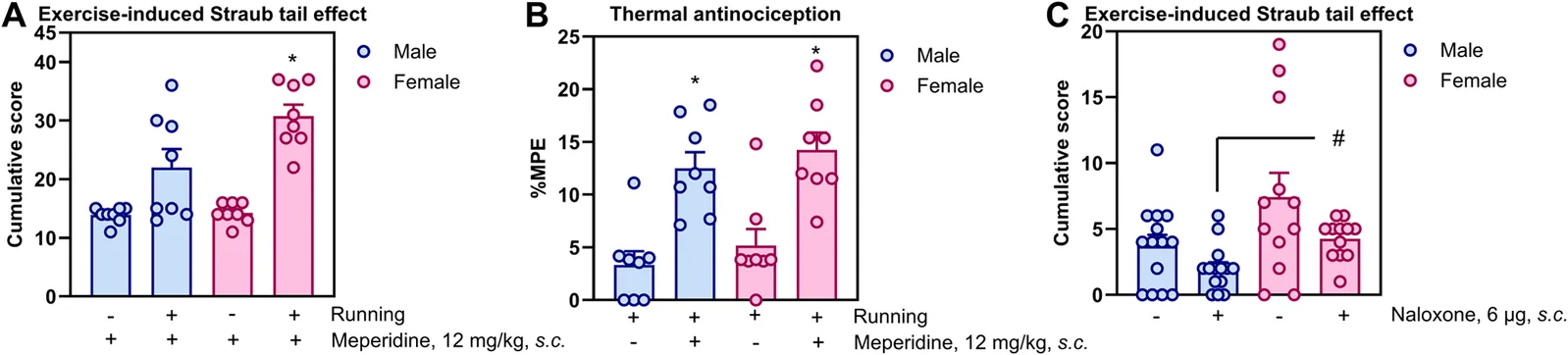
Running potentiates the meperidine-induced Straub tail response in a sex-dependent manner. Panel A shows cumulative Straub tail scores in male and female mice after meperidine 12 mg/kg, s.c., at rest and during running. A significant Sex × Exercise interaction was detected; Bonferroni post hoc analysis confirmed a significant increase in running vs resting females (*P < 0.05 vs resting condition). Panel B shows thermal antinociception (%MPE) in male and female mice after vehicle or meperidine 12 mg/kg, s.c. Both sexes showed significant antinociception (*P < 0.05 vs vehicle; Bonferroni post hoc), with no Sex × Dose interaction. Panel C shows cumulative Straub tail scores in male and female mice running after vehicle or naloxone (6 μg/kg, s.c.). Naloxone-treated females maintained higher scores than naloxone-treated males (^#^ *P* < 0.05; Bonferroni post hoc). Data are presented as mean ± SEM; *n* = 8 animals per group in panels A and B, and *n* = 12 per group in panel C. Two-way ANOVA followed by post hoc comparisons within a prespecified family of three contrasts per panel, each tested with the standard deviation of the two cells compared and Bonferroni-corrected across the three, as described in the Methods section. The rest-versus-running comparison in males is marked in Figure 1G, where it is tested alone by Welch’s *t* test (*P* = 0.037), but not in panel A, where the same comparison is corrected for the family of three (*P* = 0.069); the two are not in conflict, and we report both rather than the more favorable one.

These findings align with the emerging human evidence. Nold et al.^22^ found that high-intensity aerobic exercise did not reduce pain relative to low-intensity exercise in their sample as a whole; in an exploratory, nonpreregistered analysis, however, males with higher fitness levels showed greater exercise-induced hypoalgesia than females, an effect abolished by naloxone and mirrored by activation of the medial frontal cortex. The authors explicitly presented this sex effect as requiring confirmation. Notably, females in that study showed a stronger interaction between naloxone and pain intensity than males, suggesting that endogenous opioid engagement during exercise differs not only in magnitude but also in the specific pain circuits recruited between sexes. What the rodent and human data sets share is the presence of sex-dependent opioid involvement during exercise, rather than a common direction of effect: Nold et al.^22^ reported greater exercise-induced hypoalgesia in males, whereas running potentiated meperidine-induced Straub tail behavior more strongly in females here. Given the differences in species, end point, and pharmacological challenge, we take the human data as indicating that sex is a relevant variable in exercise-opioid interactions, not as independent confirmation of the present result.

The naloxone experiment requires a more cautious reading. Two-way ANOVA revealed significant main effects of both sex (*F*(1, 44) = 7.43, η^2^ *p* = 0.144, 1 – β = 0.76, *P* = 0.009) and treatment (*F*(1, 44) = 4.82, η^2^ *p* = 0.099, 1 – β = 0.57, *P* = 0.033), with no significant Sex × Treatment interaction (*F*(1, 44) = 0.46, η^2^ *p* = 0.010, 1 – β = 0.10, *P* = 0.499; Figure 3C). Bonferroni post hoc analysis showed that naloxone-treated females maintained higher cumulative Straub tail scores than naloxone-treated males (Hedges’ *g* = 1.27, *P* = 0.012, Bonferroni-corrected). Because the Sex × Treatment interaction was not significant, these data do not establish that naloxone was less effective in females; the difference observed under naloxone is equally consistent with the main effect of sex, females showing higher running-induced scores overall. What the experiment shows is that a sex difference persists in the presence of opioid receptor blockade. Whether this residual reflects nonopioid mechanisms recruited by exercise, such as endocannabinoid signaling, which mediates running-induced anxiolysis and analgesia in mice,^24^ serotonergic and catecholaminergic modulation of the response, both of which alter morphine-induced Straub tail behavior,^16,25^ or differential activation of the sacrococcygeus dorsalis muscle that produces the tail elevation,^14^ cannot be determined from the present design, which included no antagonists of those systems. Testing it would require selective blockade of each candidate pathway in both sexes.

The biological basis of these sex differences in opioid pharmacology remains complex and incompletely understood. Mogil et al.^21^ showed that the direction and magnitude of sex differences in nociception and morphine antinociception depend critically on genotype and strain, with all possible outcomes observed across outbred rodent populations. At the receptor subtype level, Taylor et al.^26^ reported that sex differences in κ-opioid receptor antinociception are influenced primarily by X chromosome complement rather than gonadal hormones, raising the possibility that chromosomal rather than hormonal factors also contribute to the μ-opioid dimorphism observed here during exercise, though this remains to be directly tested. Regardless of mechanism, the present findings indicate that biological sex and exercise intensity jointly modulate the behavioral response to a μ-opioid agonist in a manner that is not captured by resting-state drug assessments alone.

Two features of meperidine warrant comment. First, meperidine is no longer a first-line clinical analgesic: its analgesic efficacy is modest, and its use is complicated by serotonergic toxicity and by accumulation of the metabolite normeperidine, which produces central nervous system excitation ranging from nervousness and tremor to multifocal myoclonus and seizures. Normeperidine accumulation is favored by repeated dosing and by renal insufficiency, although renal dysfunction is not its sole determinant.^27–29^ These are liabilities of repeated administration in patients and do not bear on the single acute subcutaneous dose used here, in which meperidine served as a pharmacological probe of central μ-opioid activity rather than as a model of contemporary prescribing practice. Second, meperidine is not a selective μ-agonist. It was originally synthesized as an atropine analogue and retains anticholinergic activity; it blocks sodium channels with the molecular features of a local anesthetic; and it inhibits the reuptake of both serotonin and noradrenaline.^29^ The last of these warrants emphasis in the present context, because serotonergic compounds modulate the morphine-induced Straub tail response;^25^ a serotonergic contribution to the effects reported here therefore cannot be excluded on pharmacological grounds alone. Two observations argue against a major nonopioid contribution at the doses studied: spontaneous locomotor activity was unchanged at 8 and 12 mg/kg in both sexes (Figures 1B and 2B), arguing against a generalized stimulant action, and the Straub tail response to meperidine is reversed by opioid antagonists.^15^ Replication with a more selective μ-agonist would nonetheless strengthen the inference.

How meperidine compares with a clinically current μ-agonist can be assessed against published data obtained under closely comparable conditions. Collins et al.^30^ measured oxycodone-induced antinociception in male and female mice on a 54 °C hot plate, the temperature used here, and reported significant antinociception at 3.2, 5.6, and 10 mg/kg in both sexes, with a larger total effect in males at the highest dose. Meperidine required 12 mg/kg to produce comparable thermal antinociception in males in the present study, consistent with its lower potency. The comparison is informative in a second respect. In that study, oxycodone increased locomotor activity at 1–10 mg/kg in females and at 3–10 mg/kg in males, whereas meperidine at 8 and 12 mg/kg did not alter spontaneous locomotion in either sex here (Figures 1B and 2B). The absence of a locomotor effect of meperidine therefore does not indicate an absence of pharmacological action, since the same doses produced large thermal antinociception (Hedges’ *g* = 2.15 in males and 1.89 in females at 12 mg/kg), but reflects a difference in behavioral profile between the two drugs. For a design in which the behavioral readout is scored during treadmill running, an agonist that does not itself alter locomotion is an advantage: locomotor activation by the drug would be difficult to separate from the effect of exercise. This comparison is necessarily indirect. The two studies differ in mouse strain (outbred Swiss versus C57BL/6J), route of administration (subcutaneous versus intraperitoneal), and in the locomotor assay itself, which was recorded here over 5 min in an open field and by Collins et al. over 60 min in an activity-monitoring frame; a short-duration open field is less sensitive to late or sustained changes in activity. The open field data reported here, and the individual records from which they derive, are openly available in the repository cited above, so that this comparison can be re-examined directly.

Three limitations bear on the strength of these conclusions. The Straub tail response was scored in real time by a single observer who was aware of treatment allocation, without a second rater and without a video record from which agreement could be calculated; the design therefore cannot exclude observer bias, although the principal null findings run counter to the experimental hypothesis and the dose–response relationship is graded across five conditions in both sexes. The naloxone experiments are underpowered within each sex, and the one treatment effect that does reach significance emerges only when the sexes are pooled. And the potentiation itself was observed at only a single dose in each sex, over a single 30 min session, with a behavioral end point that indexes central μ-opioid engagement but does not measure receptor occupancy directly.

Any translational reading of these results must be cautious. The co-occurrence of opioid therapy and exercise-based rehabilitation is a documented feature of current practice: for patients already receiving opioid therapy in whom benefits outweigh risks, the CDC clinical practice guideline recommends that clinicians “work closely with patients to optimize nonopioid therapies while continuing opioid therapy”, and identifies exercise and exercise therapy as prominent nonpharmacologic modalities for back pain, fibromyalgia, and hip or knee osteoarthritis.^12^ What the present data add is a preclinical observation that acute running can modify the behavioral response to a μ-agonist, and that the magnitude of this modification differs between sexes. They do not establish that the same interaction occurs in humans; they were obtained with a drug that is no longer in routine clinical use, at a single acute dose, and with a behavioral rather than a clinical end point. We therefore present this work for what it is a single exploratory pharmacological challenge in an animal model, several steps removed from the clinical setting. Its value is to identify exercise intensity and biological sex as variables worth examining in future studies of opioid pharmacodynamics during physical activity, not to provide a basis for modifying rehabilitation protocols in patients.

## Methods

### Animals

Adult male and female Swiss mice were used in this study. A total of 212 noninbred mice, aged 8–10 weeks and weighing 46 ± 1.5 g, were obtained from the Central Animal Facility of the Federal University of Santa Catarina, Brazil. Animals were housed in groups of up to eight per cage in standard polypropylene cages containing wood-shaving bedding. The animal room was maintained under controlled environmental conditions, with a 12 h light/dark cycle, lights on at 7:00 a.m. and off at 7:00 p.m., ambient temperature of 21 ± 1 °C, and relative humidity of 50 ± 20%. Standard laboratory chow and filtered tap water were available ad libitum. Cages, bedding, and water bottles were changed three times per week. Animals were monitored regularly throughout the experimental period to ensure welfare.

Animals were randomly allocated to the experimental groups, and each mouse was considered an independent experimental unit. All animal experiments were performed following the protocols evaluated and approved by the Ethics Committee on Animal Use of the Federal University of Santa Catarina (CEUA/UFSC), Florianópolis, Brazil (Ethics Approval Number: 2036240921). Procedures complied with Brazilian Federal Law 11,794/2008 and with the guidelines of the National Council for the Control of Animal Experimentation (CONCEA). Experimental design, reporting, and animal welfare procedures followed the ARRIVE guidelines.

At the end of the experimental procedures, animals were euthanized by chemical anesthesia with ketamine/xylazine, followed by cervical dislocation. Ketamine and xylazine were administered at doses of 90 and 10 mg/kg, respectively.

### Inclusion and Exclusion Criteria

Exclusion criteria were prespecified in the approved ethics protocol: failure to habituate to the treadmill during the acclimation period, and deterioration of the body condition score. A further criterion was applied at screening for the hot plate test, in which only animals responding within 15 s at baseline were tested. In the event that no animal met an exclusion criterion at any stage, no animal failed to habituate to the treadmill, and no animal was lost after randomization, every randomized animal contributed data to the analyses reported here.

### Drugs

Meperidine hydrochloride was obtained from União Química Farmacêutica Nacional S/A, Brazil. Naloxone hydrochloride was obtained from Cristália, Brazil. Both drugs were freshly dissolved in sterile saline solution, NaCl 0.9%, immediately before use. Treatments were administered subcutaneously at an injection volume of 10 mL/kg. The subcutaneous route was chosen for consistency with the pharmacological literature on the Straub tail response, in which opioids and opioid antagonists have been given subcutaneously since the original characterization of the assay,^15^ and because it avoids the risk of inadvertent intravisceral injection inherent to the intraperitoneal route. Animals were weighed on the day of experimentation to calculate the individual dose. Behavioral testing started 15 min after drug administration.

### Vaginal Cytology

The estrous cycle stage of female mice was determined by vaginal cytology based on the predominant cell type observed in vaginal smears. Two to three consecutive vaginal lavages were collected using 20 μL of distilled water and transferred onto glass slides. Smears were air-dried at room temperature, stained with 0.1% crystal violet for 1 min, washed twice with distilled water, and air-dried again. Slides were mounted 24 h later with Eukitt mounting medium and examined under an optical microscope at 1×, 5×, and 20× magnification.^31^

Estrus cycle stages were classified according to the relative predominance of nucleated epithelial cells, cornified epithelial cells, and leukocytes. Proestrus was defined by predominance of nucleated epithelial cells, estrus by predominance of cornified epithelial cells, metestrus by similar proportions of the three cell types, and diestrus by predominance of leukocytes.^32^

Estrous cycle stage was recorded as a descriptive control variable and not as an independent variable. Females were allocated to treatment without regard to cycle stage and were tested at whatever stage they were in, so that the data describe an unselected female population rather than a defined hormonal state. Smears were collected from eight females per treatment group; in two per group, the recovered material was too sparse for the predominant cell type to be determined, leaving six classifiable smears per group. The observed distributions, for vehicle, meperidine 8 mg/kg, and meperidine 12 mg/kg, respectively, were: metestrus 0, 2, and 1; diestrus 4, 0, and 2; proestrus 1, 1, and 2; estrus 1, 3, and 1. These did not differ between groups (Fisher–Freeman–Halton exact test, *P* = 0.281), but with six females per group, the test has little power, and the result indicates only that no imbalance was detected, not that the groups were equivalent in cycle stage. The study was neither designed nor powered to examine cycle stage as a factor, and we make no claim about its influence on the responses reported here. Our own previous work shows that estrous stage affects exercise performance in mice,^31^ so stage-stratified designs would be informative; that question is left open.

### Straub Tail Response Scoring

The Straub tail response was assessed by direct observation and classified using a modified intensity score, applied as previously described.^16,25^ Scores were assigned as follows: 0, no response, with the tail below 30°; 1, tail elevation between 30° and 45° above the horizontal line; 2, tail elevation between 46° and 90°; 3, tail elevation greater than 90°; and 4, S-shaped curvature of the tail.

Scoring was performed in real time, at 3 min intervals, by a single trained observer (T.N.), who was aware of the treatment allocation. The experiment was therefore not blinded; no second observer scored any part of the material; and no video record was retained from which inter-rater agreement could be calculated retrospectively. We report this rather than justify it: the angular criteria above are explicit and were applied uniformly, and the raw per-animal scores are openly deposited, but neither substitutes for blinded assessment. Two features of the data bear on the direction of any resulting bias. The dose–response relationship in Experiment 1 is graded and monotonic across five treatment conditions in both sexes, a pattern less readily produced by expectancy than a single group difference; and the principal null results, namely the absence of an effect of naloxone in either sex, the absence of a group × time interaction at either dose, and the failure of the 8 mg/kg comparison to reach significance, run counter to the experimental hypothesis, whereas observer bias would be expected to act in its favor. Confirmation of the present findings requires video-recorded scoring by an observer blinded to treatment, with a second rater and a reported measure of agreement.

### General Behavioral and Exercise Procedures

Before behavioral and exercise procedures, mice were habituated to the experimental room in their home cages for 1 h. All experiments were performed during the light phase of the circadian cycle, between 8:00 a.m. and 2:00 p.m., under controlled environmental conditions, with room temperature maintained at 22 ± 1 °C, illumination at 10–12 lx, and background noise below 58 dB.

For the Straub tail assessment, mice were individually habituated to transparent plastic cages for three consecutive days before testing. The habituation period lasted for 20 min on the first day, 40 min on the second day, and 60 min on the third day. This procedure was used to reduce novelty-related stress before the behavioral observation.

Treadmill running was performed on a 10-lane motorized treadmill for mice (model TM-60 USB; Bonther, Ribeirão Preto, SP, Brazil), with an exercise area of 40 × 5 × 10 cm^3^ per lane. For treadmill experiments, mice were first habituated individually to the treadmill lane for 5 min 1 day before treadmill acclimation. Treadmill acclimation was then performed for three consecutive days, with 24 h between sessions. The protocol consisted of 10 min at 5 m/min on the first day, 10 min at 10 m/min on the second day, and 10 min at 15 m/min on the third day. A low-intensity electrical stimulus of 0.2 mA (10% of the maximum output of this apparatus, which is adjustable from 0 to 2 mA) was delivered through the rear grid only when an animal stopped running. For reference, this intensity is below that required to establish aversive memory in the step-down inhibitory avoidance task, in which 0.35 and 0.5 mA at 62 and 125 Hz, but not 0.20 mA at any frequency tested, supported learning in rats,^33^ and it is below the range of 0.25–1.0 mA used to establish Pavlovian fear conditioning in mice.^34^ Neither comparison establishes the stimulus used here as innocuous; both indicate only that it is weaker than intensities selected to be aversive, and the inhibitory avoidance data were obtained in rats. After a 48 h resting period, animals were submitted to the treadmill running experiments.

### Open Field Test

Spontaneous locomotor activity was assessed in an open field test. Mice were individually placed in an unfamiliar circular open field arena and allowed to explore freely for 5 min. The arena was 60 cm in diameter and 50 cm in height. Animal movement was recorded by a camera positioned 1 m above the apparatus, and locomotor activity was analyzed using the ANY-maze video-tracking system. The apparatus was cleaned with 20% ethanol between animals.^35^

### Hot Plate Test

Thermal antinociception was assessed using the hot plate test after the treadmill running session, in all groups including vehicle, so that sexes and doses are compared under a common exercise condition. Before the experiment, mice were screened for baseline thermal sensitivity, and only animals that responded within 15 s were included. Mice were individually placed on a hot plate apparatus maintained at 54 ± 1 °C. The latency to hind paw licking was recorded as the nociceptive end point. Animals were immediately removed from the plate after paw licking. A cutoff time of 30 s was used to prevent tissue injury.

The percentage of maximum possible effect (%MPE) was calculated for each mouse using the following formula: %MPE = [(post-treatment latency – baseline latency)/(cutoff latency – baseline latency)] × 100, where baseline latency was obtained from predrug measurements and the cutoff latency was set at 30 s.^36^

### Cardiopulmonary Exercise Testing

Cardiopulmonary exercise testing was performed to evaluate the effects of meperidine on maximal oxygen consumption and exercise performance. Oxygen consumption (V̇O_2_) and respiratory exchange ratio (RER; V̇CO_2_/V̇O_2_) were measured using a single-lane metabolic chamber coupled to a motorized treadmill (Bonther, Ribeirão Preto, SP, Brazil), a separate apparatus from the multilane treadmill used for the running sessions. Animals were habituated to the treadmill system following the same acclimation protocol described in the General Behavioral and Exercise Procedures section. After a 48 h resting period, animals underwent cardiopulmonary exercise testing. The test started at 5 m/min, and treadmill speed was increased by 5 m/min every 3 min until exhaustion. The treadmill was set at an inclination of 1.7°, and the same 0.2 mA stimulus was available. The primary criterion for maximal exertion was an RER ≥ 1.0. As secondary indicators, experimenters monitored for a plateau tendency in the V̇O_2_ curve and for exhaustion-related behavior, that is, consistent refusal to continue running despite the 0.2 mA stimulus. These complementary signs were not used as formal end points but provided supportive information, following the procedure of Alves et al.^37^

### Experimental Design

#### Experiment 1: Meperidine Dose–Response and Time Course

To characterize the time course and dose–response profile of the Straub tail response, mice received subcutaneous injections of saline or meperidine at doses of 8, 12, 32, or 96 mg/kg. These doses were selected to span the range over which meperidine had previously been characterized in the Straub tail assay.^15^ Fifteen minutes after treatment, animals were individually placed in transparent plastic cages and observed at 3 min intervals for 60 min. The Straub tail response was recorded and scored as described above.^15^

Based on the dose–response profile obtained in this experiment, the submaximal doses of 8 and 12 mg/kg were selected for the subsequent treadmill experiments.

#### Experiment 2: Submaximal Doses of Meperidine Combined with Treadmill Running

To determine whether treadmill running modifies the Straub tail response induced by submaximal doses of meperidine, mice received meperidine at 8 or 12 mg/kg, s.c., 15 min before treadmill running. Animals were then placed on the treadmill and subjected to a 30 min running session at 20 m/min. The Straub tail response was recorded at 3 min intervals throughout the running session and scored as described above. The treadmill speed of 20 m/min was selected based on an independent preliminary experiment and corresponded to 72 ± 15% of V̇O_2_ max in males and 67 ± 15% in females, calculated for each animal as the V̇O_2_ measured at that speed divided by its own V̇O_2_ max; two males reached V̇O_2_ max below 20 m/min and are not included in that mean.^37^

#### Experiment 3: Naloxone and Treadmill-Induced Straub-like Response

To investigate whether treadmill running induces a Straub-like response involving endogenous opioid signaling, mice received naloxone at 6 μg/kg, s.c., or vehicle 15 min before the running session. Animals were then subjected to treadmill running, and the Straub tail response was recorded at 3 min intervals for 30 min and scored as described above.^15^

### Statistical Analysis

Data are presented as mean ± standard error of the mean (SEM), and the sample size (n) represents the number of animals per experimental group.

The sample size was not derived from an a priori power calculation. Groups of eight animals were adopted from our previous studies using the same treadmill and behavioral procedures^31^ and from the group size adopted in the most recent pharmacological characterization of the assay;^15,16^ the naloxone experiment used 12 animals per group. The approved ethics protocol authorized 552 animals; 212 were used, and the numbers reported here are those actually studied. Because the design was not powered in advance, achieved power (1 – β) was computed post hoc for every omnibus test and is reported alongside each result, so that the evidential weight of each comparison, in particular of the comparisons that did not reach significance, can be judged directly rather than inferred from the *P* value alone.

For continuous outcomes, data distribution was assessed using the Shapiro–Wilk test. Five of the 30 groups entering parametric tests deviated from normality (*W* = 0.775–0.822, *P* = 0.015–0.049). Parametric analyses were retained because the design is balanced and the deviations were confined to a minority of groups. Rank-based analyses of the same comparisons agree with the parametric results throughout, with two exceptions that we note rather than leave for the reader to find in the resting dose–response the vehicle group scored zero in every animal and every observation, so that complete ties deprive a rank test of power and the 8 mg/kg comparison is not detected by it; and the potentiation by running at 12 mg/kg in males, significant by Welch’s test (*P* = 0.037), falls just short by the Mann–Whitney test (*P* = 0.060). The per-animal data are deposited so that either analysis can be reproduced. Comparisons between two groups were performed using Welch’s unpaired *t* test. Comparisons involving more than two groups were performed using one-way ANOVA followed by Tukey’s post hoc test. For time-course data obtained from two groups under repeated measurements, a mixed ANOVA model was applied, with group as the between-subjects factor and time as the within-subjects factor; Bonferroni correction was applied for post hoc comparisons across time bins. For the dose–response time-course experiment involving more than two groups with repeated measurements, two-way repeated-measures ANOVA was used, followed by Bonferroni post hoc analysis. Comparisons involving two crossed between-subjects factors (Sex × Exercise, Sex × Dose and Sex × Treatment) were performed by two-way independent ANOVA computed with Type III sums of squares. Post hoc comparisons were restricted to a prespecified family of three contrasts per panel (the effect of the second factor within each sex, and the difference between sexes under the active condition), and each was tested with the standard deviation of the two cells compared rather than the pooled error term, with Bonferroni correction across the three. This choice is deliberate: cell variances were heterogeneous in the Sex × Exercise and Sex × Treatment analyses (Brown–Forsythe *P* < 0.001 and *P* = 0.003, with cell standard deviations spanning 1.4–8.9 and 1.5–6.4 respectively), so a pooled error term would misstate the precision of individual contrasts. The same heterogeneity qualifies the omnibus two-way tests in those two analyses, which do assume a common error variance; we report them with that limitation stated rather than omitted. The distribution of estrous cycle stages across treatment groups was compared with the Fisher–Freeman–Halton exact test, the counts being too small for a chi-square approximation to be valid.

For primary between-group comparisons of the Straub tail response, the cumulative score was calculated for each animal as the sum of scores across the observation period and analyzed using Welch’s unpaired *t* test. For the naloxone experiment, a one-tailed test was applied based on the directional hypothesis, specified before the experiment, that opioid receptor blockade could reduce but not increase a response mediated by endogenous opioids. Because a one-tailed test is the more permissive choice, both tailed values are reported for those two comparisons; neither reaches significance under either convention.

Effect sizes were calculated as Hedges’ *g* for two-group comparisons and partial eta squared (η^2^ *p*) for ANOVA models. Hedges’ *g* was used as a bias-corrected standardized mean difference, appropriate for small sample sizes, and interpreted as small, 0.2; medium, 0.5; and large, ≥0.8. Partial η^2^ *p* values were interpreted as small, ≥0.01; medium, ≥0.06; and large, ≥0.14. Statistical power (1 – β) was estimated for all omnibus tests. Statistical significance was set at *P* < 0.05.

## Data Availability

All raw data and statistical analysis scripts are publicly available at the Open Science Framework repository under a CC BY 4.0 license.^38^

## Abbreviations

ANOVA: analysis of variance
ARRIVE: Animal Research: Reporting of In Vivo Experiments
CEUA: Ethics Committee on Animal Use
CONCEA: National Council for the Control of Animal Experimentation
% MPE: percentage of maximum possible effect
PET: positron emission tomography
RER: respiratory exchange ratio
s.c.: subcutaneous
SEM: standard error of the mean
V̇O_2_ max: maximal oxygen uptake
